# On genomics, kin, and privacy

**DOI:** 10.12688/f1000research.3817.1

**Published:** 2014-03-31

**Authors:** Amalio Telenti, Erman Ayday, Jean Pierre Hubaux

**Affiliations:** 1 Department of Laboratories, University Hospital of Lausanne, Lausanne, Switzerland; 2 Laboratory for Communications and Applications Laboratory, École Polytechnique Fédérale de Lausanne, Lausanne, Switzerland

## Abstract

The storage of greater numbers of exomes or genomes raises the question of loss of privacy for the individual and for families if genomic data are not properly protected. Access to genome data may result from a personal decision to disclose, or from gaps in protection. In either case, revealing genome data has consequences beyond the individual, as it compromises the privacy of family members. Increasing availability of genome data linked or linkable to metadata through online social networks and services adds one additional layer of complexity to the protection of genome privacy.  The field of computer science and information technology offers solutions to secure genomic data so that individuals, medical personnel or researchers can access only the subset of genomic information required for healthcare or dedicated studies.

## Introduction

The recent authorization of a sequencing platform for clinical use by the Food and Drug Administration will expand and accelerate the use of genetic information in medical care ^1^. Progress is particularly impressive in the deployment of sequencing tools for neonatal diagnostics ^2^. Commoditization of genome-wide genotyping and sequencing is happening as rapidly outside of the medical setting – prominently through companies offering “direct to consumer” (DTC) services. There is full awareness of the need to protect these data ^1^ – while simultaneously supporting their use in research ^3^. Here, we discuss how protection of genome data from medical and non-medical sources needs to be reframed considering the mutual implications of personal decision, online social networks and consequences to relatives.

### On personal decisions

Paradoxically, genomics is an attractive field for individual or collective altruism – many people are willing to place their genome data in the public domain, and to actively engage in genomic research. The academic community is also calling for definitive actions to support global data-sharing ^3^. Many research participants count on the protection of their identity. However, current strategies have proven insufficient to stop sophisticated attacks on genetic data. A recent study ^4^ demonstrated the feasibility of re-identifying DNA donors from a public research database by using information available from popular genealogy websites. Attackers can also take advantage of gaps in the protection of other sources of data, for example census and voter lists, hospital insurance reports, and increasingly, from online social networks (see below). Genome data in the wrong hands could have undesirable consequences: from discrimination, or release of paternity, ancestry or other data that the participant did not intend to be public, to more prosaic usages such as targeted advertisements based on genome information.

### Genome and online social networks

**Figure 1.**
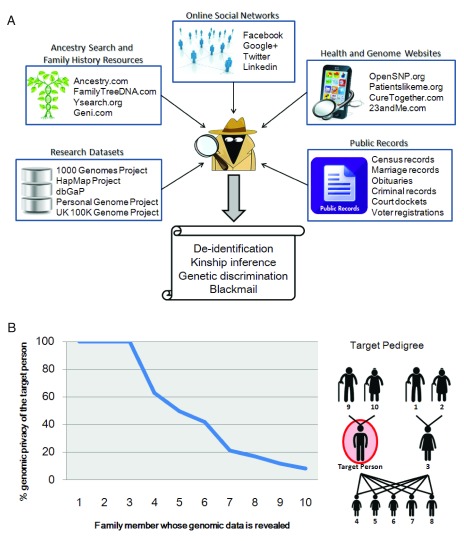
Attacks on genomic privacy. ( **A**) Multilayer attacks using data from genomic and non-genomic platforms. An attacker can obtain the anonymized genomic data of an individual from one of the genome data websites (e.g., openSNP.org). Then, the attacker can de-anonymize the owner of the genome (i.e., learn his/her identity) by matching his/her phenotypic, demographic and administrative information (e.g., profile picture, age, gender, ZIP code) across the individual’s online social network profile. Once the individual is de-identified, the attacker can also determine his/her family members from a family history resource (e.g., ancestry.com) and infer the genomic data of family members from the individual’s retrieved genome. For example, owners of some genomes uploaded to openSNP can be de-anonymised using their Facebook profiles. For 6 individuals who publicly revealed the names of some of their relatives on Facebook, 29 familial relationships could be identified ^9^. ( **B**) Decrease in genomic privacy of the target person (circled in red) when the genomes of his family members are gradually revealed. The health privacy of family members can be quantified. For example, two single nucleotide polymorphisms (rs7412 and rs429358) of the Apolipoprotein E ( *ApoE*) gene are associated with increased risk for Alzheimer's disease. The identification in several members of the pedigree of a carrier status for those risk alleles can reveal the *ApoE4* status of the target person to the attacker.

 Online social platforms are convenient sites for posting data but they are susceptible to “multilayer attacks”: the possibility to simultaneously aggregate data from online social networks (e.g., Facebook), health related websites (e.g., patientslikeme.com), platforms for sharing genome data (e.g., OpenSNP.org), family history resources (e.g., ancestry.com), research datasets (e.g., 1000 Genomes Project), and public records (e.g., voter registration forms) can help an attacker de-anonymize the owner of an anonymized genome and/or infer the genomic data of his/her family members. We illustrate in Figure 1A the feasibility and ease of cross-identification of a given individual across various genetic and non-genetic platforms, including the reconstitution of parts of the family pedigree.

### On kinship issues

Kin aspects of genomics were well publicized by the recent controversy regarding the public release of the genome of Henrietta Lacks (August 1, 1920 – October 4, 1951). HeLa, a cell line established from Lacks, has been used for decades in research laboratories world-wide. Recently, HeLa cells were sequenced and the genome data posted online without the consent of her relatives, who subsequently complained that this accounted to revealing private information about the family. The multilayer attacks mentioned above can reconstruct phylogenies from revealed genomes and open the door to genetic prediction of family members. The amount of kin privacy lost from such attacks can be precisely estimated ( Figure 1B). As more individuals will have their genome sequenced or genotyped in coming years, the loss of privacy of family members through multilayer attacks will increase if no action is taken.

### Solutions from computer science

There is little doubt that genome privacy will be challenged – in particular if the medical establishment relies solely on legal deterrents and conventional protection of stored data, or if it resorts to ineffective deidentification and anonymization of genome data shared for the purpose of research. However, personal genetic tests and genomic research are possible without jeopardizing the genomic privacy of the individual or of family members. In particular, IT security provides a trove of solutions. These include using efficient cryptographic techniques for privacy-preserving personalized medicine ^5, 6^, and for genomic research ^7^. With such approaches, genomic data are always stored in encrypted form and medical personnel or researchers can access only the subset of genomic information required for healthcare or dedicated studies. Similarly there are obfuscation-based solutions ^8^ to use genomic data in research settings in a privacy-preserving way.

Some genome researchers may be tempted to belittle the threat raised by the possible leakage of genomic data. This is a mistake, because progress in genetics is likely to make these data more and more meaningful. In addition, if it appears that genomic data are not properly protected, people could start distrusting genetics, with negative consequences for the progress of medicine. Protection needs to consider both the interest of the individual and of relatives. It is important to learn from errors in Internet security over the last decades. In that field, tools and solutions are often lagging behind threats.

The first meeting exclusively dedicated to genomic privacy took place in October 2013 at the Leibniz Center for Informatics in Dagstuhl, Germany ( http://www.dagstuhl.de/13412). As one of the outcomes, the community set up a web site reporting the efforts and progress on this topic: https://genomeprivacy.org/. Notably, this site contains the list of research groups active in this field, as well as basic information to facilitate the understanding of this novel field. It is our conviction that by pooling together the skills of geneticists, law scholars, ethicists and computer scientists, we are still in time to strike an appropriate balance between accessibility to genome data and their protection.
