# Supplementary material for: On genomics, kin, and privacy
Source: F1000Res. 2014 Mar 31;3:80. [Version 1] doi: 10.12688/f1000research.3817.1 (PMC4168742; doi:10.12688/f1000research.3817.1)
Supplement: Peer review history

# Peer review: Referee response for version 1

**DOI:** 10.5256/f1000research.4089.r5895

**Published:** 2014-08-29

**Authors:** Florian Kerschbaum

**Affiliations:** 1 SAP, Karlsruhe, Germany

This article raises a very important issue: the difficulty of providing privacy for genetic information in the light of inheritance. I cannot stress enough how important this aspect is, since it requires data protection measures, such as the mentioned cryptographic or obfuscation-based approaches, to be utterly restrictive. The article gives clear examples where -- intentionally or unintentionally -- leaked information allowed harmful inferences. The authors combine information from public genomes and social networks to infer information about people who have not released any information about their genes. These examples should be taken very, very seriously, since they are raised only at the very beginning of the scientific development. As the paper argues we can expect the use of genomics to significantly grow. Genetic information can prove at least as harmful as location information provided by cell phones, but it is impossible (for now) to change it. It is therefore a scientific and societal challenge to protect genomic information much better than we protect our information in current telecommunication networks. The article references excellent works from the computer security community that can certainly provide a guiding direction, but even these mechanisms need to necessarily leak some information about the genomes. I hope that medical and computer science researchers will take the challenge described by the article seriously and look for mechanisms that control the entire information in all medical or non-medical information system based on direct or indirect genomic data.

I have read this submission. I believe that I have an appropriate level of expertise to confirm that it is of an acceptable scientific standard.

# Peer review: Referee response for version 1

**DOI:** 10.5256/f1000research.4089.r5889

**Published:** 2014-08-29

**Authors:** Xiaoqian Jiang

**Affiliations:** 1 Division of Biomedical Informatics, University of California, San Diego, La Jolla, USA

This is a timely commentary on privacy, kin, and genomics. Today, many gene donators are still ignorant of the potential impact of information leakage to the family when their genome data are made public. This problem is becoming more critical as the younger generation reveals themselves and family members on online social networks and open family history resources made it possible to link individuals. I found this topic to be extremely important.

I have read this submission. I believe that I have an appropriate level of expertise to confirm that it is of an acceptable scientific standard.

# Peer review: Referee response for version 1

**DOI:** 10.5256/f1000research.4089.r4304

**Published:** 2014-04-14

**Authors:** XiaoFeng Wang

**Affiliations:** 1 Centre for Security Informatics, School of Informatics and Computing, University of Indiana at Bloomington, Bloomington, IN, USA

This paper discusses the challenge of protecting human genome data, particularly its unique feature in that one’s DNA data can be used to infer the private health information of those genetically related to them. The authors talk about the conflict between the perception that the decision on releasing one’s DNA materials is personal, and how it can actually impact on the privacy of their kin. They further sketch a technique for quantifying such an information leak, and demonstrate that the threat is realistic, given the de-anonymization attack that can happen through the booming online social networks. I feel that this article provides useful information for raising the awareness of the uniqueness and significance of genome privacy. This, hopefully, will lead to a broad, in-depth conversation among genomics researchers, security and privacy researchers, bioethics experts, genomics industry, policy makers and the public on how to effectively regulate the dissemination of human DNA data to facilitate scientific research, without undermining DNA donors’ privacy and well-being.

I have read this submission. I believe that I have an appropriate level of expertise to confirm that it is of an acceptable scientific standard.
